# Letter to the Editor Regarding “Impact of Anisotropic Conduction and Premature Atrial Contraction on the Fractionated Atrial Potentials”

**DOI:** 10.1002/joa3.70262

**Published:** 2025-12-30

**Authors:** Hamza Ibrahim, Laraib Riaz, Muqeet Hasnain

**Affiliations:** ^1^ Allama Iqbal Medical College Lahore Pakistan; ^2^ Sargodha Medical College Sargodha Pakistan

To the Editor,

We read with interest the article titled “Impact of anisotropic conduction and premature atrial contraction on the fractionated atrial potentials” [1]. They report that premature atrial contractions (PACs) shift fractionated atrial potentials (FAPs) away from the right and mid anterior walls toward the left anterior, left inferior, and lateral walls, emphasizing the impact of extrastimuli direction on atrial substrate characterization.

In contrast, Heida et al., using intraoperative high‐resolution epicardial mapping during spontaneous PACs in 228 patients, found no clear increase in conduction heterogeneity or fractionation in regions like Bachmann's bundle or pulmonary vein area that would support a substantial PAC‐induced FAP burden [2]. Their quantification of local directional heterogeneity revealed that while PACs modestly increased LDH, they did so uniformly and did not reproduce the dramatic regional redistribution seen by Toyama and Kumagai. This raises the question of whether PACs truly alter FAP distribution in the manner proposed by the recent study.

Moreover, Hirokami et al. studied fractionated signal areas in the atrial muscle during pacing and premature extrasystoles and concluded that the effect of premature stimuli direction on fractionation patterns was minimal, suggesting that the directionality emphasized by Toyama and Kumagai may be overinterpreted [3].

A further layer of contradiction comes from two additional studies. Teuwen et al. [4] used high‐resolution epicardial mapping to compare conduction during sinus rhythm and atrial extrasystoles (AES) in over 160 patients; they found that decreases in conduction velocity and increases in delay occurred primarily in cases of aberrant wavefronts or breakthrough AES, but the degree of prematurity itself did not consistently correlate with increased conduction block or fractionation, indicating that not all PACs cause enhanced FAP burden [4]. Additionally, modeling and optical mapping work in canine atria by Roberts‐Thomson et al. demonstrated that anisotropic conduction properties and resulting fractionated electrograms were highly dependent on tissue structural geometry and pacing rate rather than PAC direction per se—suggesting a more complex interplay than Toyama and Kumagai's direction‐based hypothesis allows [5].

Taken together, these contradictory findings highlight that while Toyama and Kumagai's work provides valuable insight into how anisotropic conduction and extrastimuli may affect FAP distribution, other clinical and modeling data challenge the magnitude and regional specificity of those effects—emphasizing the need for standardized high‐density mapping and controlled comparisons between spontaneous and paced PACs to resolve these discrepancies.

## Conflicts of Interest

The authors declare no conflicts of interest.
